# The Role of *AtMUS81* in Interference-Insensitive Crossovers in A. thaliana

**DOI:** 10.1371/journal.pgen.0030132

**Published:** 2007-08-10

**Authors:** Luke E Berchowitz, Kirk E Francis, Alexandra L Bey, Gregory P Copenhaver

**Affiliations:** 1 Department of Biology, The University of North Carolina at Chapel Hill, Chapel Hill, North Carolina, United States of America; 2 The Carolina Center for Genome Sciences, The University of North Carolina at Chapel Hill, Chapel Hill, North Carolina, United States of America; University of Chicago, United States of America

## Abstract

*MUS81* is conserved among plants, animals, and fungi and is known to be involved in mitotic DNA damage repair and meiotic recombination. Here we present a functional characterization of the Arabidopsis thaliana homolog *AtMUS81,* which has a role in both mitotic and meiotic cells. The *AtMUS81* transcript is produced in all tissues, but is elevated greater than 9-fold in the anthers and its levels are increased in response to gamma radiation and methyl methanesulfonate treatment. An *Atmus81* transfer-DNA insertion mutant shows increased sensitivity to a wide range of DNA-damaging agents, confirming its role in mitotically proliferating cells. To examine its role in meiosis, we employed a pollen tetrad–based visual assay. Data from genetic intervals on Chromosomes 1 and 3 show that *Atmus81* mutants have a moderate decrease in meiotic recombination. Importantly, measurements of recombination in a pair of adjacent intervals on Chromosome 5 demonstrate that the remaining crossovers in *Atmus81* are interference sensitive, and that interference levels in the *Atmus81* mutant are significantly greater than those in wild type. These data are consistent with the hypothesis that *AtMUS81* is involved in a secondary subset of meiotic crossovers that are interference insensitive.

## Author Summary

Meiosis is a specialized type of cell division in which one diploid progenitor cell divides into four haploid cells that are subsequently used for fertilization during sexual reproduction. During meiosis, chromosomes pair, synapse, and exchange genetic information, all of which are required for proper chromosome segregation during subsequent stages. Failure to properly segregate meiotic chromosomes often leads to genetic defects such as aneuploidy. Using the model plant *A. thaliana,* we have developed a powerful system for the visual analysis of meiotic recombination directly in the pollen, in which the four products of individual meioses are fused together in a tetrad. We have used this system to characterize the gene *AtMUS81* and show that *Atmus81* mutants have a moderate reduction in meiotic crossovers and are sensitive to a wide range of DNA-damaging agents. Importantly, the remaining crossovers in *Atmus81*still exhibit crossover interference, a phenomenon whereby one crossover inhibits the occurrence of other nearby crossovers. Our results suggest that *AtMUS81* mediates a subset of meiotic recombination events in *Arabidopsis* that are insensitive to crossover interference.

## Introduction

During meiotic prophase I, homologous chromosomes pair, synapse, and exchange genetic information (via crossing over or gene conversion), all of which are required for proper chromosome segregation during the subsequent stages of meiosis, in which haploid gametes are produced from diploid progenitor cells. Extensive genetic and molecular data from the budding yeast Saccharomyces cerevisiae has led to the double-strand break repair model of meiotic recombination, in which chromosomes are subjected to programmed double-strand breaks [1–3]. In all sexually reproducing organisms studied to date, these breaks are dependent on Spo11p and are resolved leading to either crossovers (COs) or noncrossovers [4]. In most organisms, COs are distributed nonrandomly such that one CO event inhibits the chances of another nearby event and each chromosome pair usually has at least one crossover. The term used to describe this phenomenon is CO interference [5].

Statistical and experimental evidence suggests that A. thaliana, like yeast and humans, has two recombination pathways: one that exhibits crossover interference and another that does not [6–9]. This is in contrast to organisms such as Caenorhabditis elegans and Drosophila melanogaster, in which all COs are thought to be subject to interference [10,11]. In the organisms studied to date with both interfering and noninterfering COs, the majority of events are thought to be generated by the primary, interference-sensitive pathway. In both S. cerevisiae and *A. thaliana,* several genes active in the interference-sensitive pathway such as the *MSH4/MSH5* heterodimer [12] and the DNA helicase–encoding *MER3* [13] have been identified. In *A. thaliana,* disruption of these genes causes a reduction of approximately 85% of COs [6,14]. Analysis of the distribution of the residual chiasmata in *msh4/msh4* meiocytes has led to the suggestion that the remaining COs are processed by a secondary pathway that is not subject to interference. We report here on the mitotic and meiotic characterization of *AtMus81*(At4g30870), which strongly suggests a role for this gene in an interference-insensitive crossover pathway in A. thaliana.

Our lab has developed a unique pollen-based visual assay for meiotic recombination in A. thaliana that has facilitated these investigations [15,16]. This assay system is based on a series of transgenic lines, each carrying a gene encoding either a red, cyan, or yellow fluorescent marker protein excitable by different wavelengths of light. Transcription of these markers is directed by a post-meiotic pollen-specific promoter (LAT52) in the mutant *qrt1* background that produces tetrads of meiotically related pollen grains [17,18]. We constructed visually assayable genetic intervals by crossing lines carrying linked markers. Lines carrying two or more markers of different colors on the same chromosome produce tetrads that segregate the marker genes (and thus the proteins they encode) in patterns that reflect whether or not a recombination event has happened between them. Using this system, we can detect CO events directly in the gametes, and through the construction of double intervals delineated by three colors, we can assay CO interference. We used this system to assay the meiotic recombination phenotypes of the *Atmus81, Atmsh4,* and double mutants. We have also monitored production of fluorescent protein (or lack thereof) in homozygous constructs to quantify pollen viability.

The methyl methansulfonate UV sensitive (*MUS81*) gene was originally identified in S. cerevisiae in a two-hybrid screen for protein products that interact with the recombination factor Rad54p [19]. It was independently isolated in a screen for genes that are essential in *SGS1* and *TOP3* null backgrounds [20]. *Mus81* mutants show sensitivity to DNA-damaging agents in yeast [19] and in mammals [21]. In *S. cerevisiae,* mutant alleles of *MUS81* reduce, but do not eliminate, meiotic recombination. In these mutants, COs are reduced 1.1- to 1.8-fold and the residual COs are interference sensitive [8]. This is in contrast to other meiotic mutants such as *msh4,* in which the reduction in COs is greater, and the remaining COs are interference insensitive. This has led to the suggestion that in *S. cerevisiae,* there are at least two distinct classes of COs: interference-sensitive COs that require *MSH4/MSH5* and interference-insensitive COs that require *MUS81* [8]. Double mutants in both *msh4* and *mus81* in S. cerevisiae result in a severe reduction in COs, reinforcing the two-pathway model [22]. But even this combination, which reduced COs by 13- to 15-fold, had some residual CO activity, implying a possible third pathway in this organism [22].

Not all organisms have both interfering and noninterfering COs. In the yeast *Schizosaccharomyces pombe,* an organism that does not have CO interference, *MMS4/MUS81* mutants show a complete lack of COs [23]. Conversely, in the nematode *C. elegans,* an organism that shows complete interference, such that each homolog pair gets exactly one CO per meiosis, *MSH4* mutants show a complete lack of COs [24]. D. melanogaster presents another interesting case in which all COs are interference sensitive and *MUS81* mutants show no reduction in COs, but do show sensitivity to DNA-damaging agents, suggesting a role confined to the mitotic cycle (J. Sekelsky, personal communication).

In many organisms, Mus81p interacts with another protein to form a heterodimer, which is essential for its function. In *S. cerevisiae,* it is Mms4p, in *S. pombe,* it is Eme1p, and in *D. melanogaster,* the interacting partner is Mms4p [20]. In vitro studies using the fungal or human heterodimer have shown that this complex can cleave 3′ flaps and collapsed replication forks [25]. Recent evidence from S. pombe suggests a role for Mus81-Eme1 in the resolution of single Holliday junctions (HJs), which may be the primary recombination intermediates in this organism [26]. This finding is supported by the previous observation that, in *S. pombe,* expression of the bacterial HJ resolvase RusA can partially suppress the *mus81* mutant phenotype [23]. Interestingly, studies in S. cerevisiae show that the role of Mus81p as the essential HJ resolvase is not universal. In budding yeast, double (not single) Holliday Junctions (dHJs) may be the primary recombination intermediate, and expression of RusA failed to suppress the *mus81* meiotic phenotype. Physical analysis of the *S. cerevisiae mus81* deletion mutant is not consistent with a HJ resolution defect, as dHJs are processed; however, the kinetics are delayed about 2 h. Notably, dHJ intermediates are reduced in the *mms4* mutant background [8].

*S. cerevisiae mus81* mutants show synthetic lethality with *sgs1,* a helicase in the RecQ family. Intriguingly, this lethality is dependent on the *RAD52* double-strand break repair pathway [25]. A similar synthetic lethality was demonstrated in *A. thaliana,* in which *Atmus81* mutants are also synthetically lethal with mutants of the A. thaliana homolog of *SGS1: AtRecQ4A*. *AtMus81* mutants were also shown to be sensitive to the DNA-damaging agents mitomycin C and methyl methanesulfonate (MMS), confirming a mitotic role for *AtMUS81* as well [27]. In this study, we show that this sensitivity phenotype extends to other damaging agents and results in an increase in *AtMUS81* transcript levels.

A previous study found that *Atmus81* mutants do not have a detectable meiotic defect [27]. Using our visual assay for recombination, we have found that the *Atmus81* mutant has a moderate meiotic defect in the form of reduced COs. We also observed an elevation in the expression of the *AtMUS81* transcript in tissue types undergoing meiosis and decreased pollen viability in the *Atmus81* mutant. Importantly, interference is stronger in the *Atmus81* mutant, an observation that is consistent with the role of *AtMus81* in a secondary interference-insensitive CO pathway in A. thaliana.

## Results

### Isolation of the *AtMus81* Gene (*At4g30870*) and Identification of a Transfer-DNA Insertion Mutant

The A. thaliana homolog of *MUS81, AtMUS81* (At4g30870), was identified using a BLAST search using S. cerevisiae (2e−19) and S. pombe (3e−25) sequences. This search also produced a second intriguing hit: At5g39770 (3e−12, using *S. cerevisiae MUS81*) which is annotated in the MIPS database as *MUS81*-like. However, this sequence is thought to represent a nonfunctional pseudogene [27]. Using reverse-transcriptase PCR (RT-PCR) with internal primers as well as 5′ and 3′ rapid amplification of complementary ends (RACE) to amplify the ends, we confirmed the full-length cDNA sequence published by Hartung et al. with one small difference [27]. The sequence of the molecule obtained from our 5′ RACE was 5 bp shorter than what is reported in Genbank (http://www.ncbi.nlm.nih.gov/gquery/gquery.fcgi). This could be explained by two alternate transcriptional start sites, a feature of the *MUS81* transcript that was recently observed in the Oryza sativa homolog, *OsMUS81* [28]. Apart from this 5-bp difference, a composite molecule that matched the published sequence was constructed using our amplification products.

### Identification of Transfer-DNA Insertion Mutants in AtMUS81

**Figure 1:**
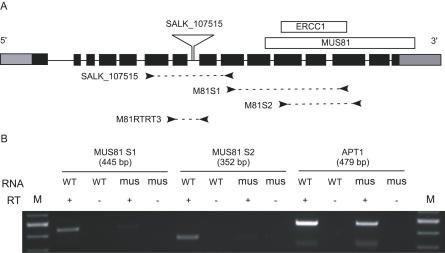
The *AtMUS81* Gene Structure, T-DNA Insertion Mutant, and Expression (A) An illustration of the *AtMUS81* (At4g30870) locus showing the exon/intron organization of *AtMUS81*. Solid boxes represent transcribed regions including protein coding (black) and untranslated regions (gray). The T-DNA insertion site for the mutant used in this study is shown. Conserved domains are shown above. Below are products for the following primers that were used for genotyping (M81_F/M81_R), RT-PCR (M81S1_F/M81S1R and M81S2_F/M81S2_R), and real-time qPCR (M81RTRT3_F/M81RTRT_R). (B) Whole seedling (10-d) RT-PCR of wild type and the *Atmus81* mutant. Primers (S1 and S2) downstream of the T-DNA insertion site were used in the RT-PCR reaction with and without reverse transcriptase (RT) using RNA from wild-type (WT) and mutant (mus) plants. The *APT1* transcript was used as a control.

The SALK laboratories SIGnAL database of transfer DNA (T-DNA) insertions contains two intronic T-DNA insertions within the open reading frame of *AtMUS81* (http://signal.salk.edu/cgi-bin/tdnaexpress) SALK_107515, and SALK_113F11 (Figure 1A). We used the former, as we were able to show that this is a bona fide insertion using PCR with primers spanning the insert (Figure 1A). Hartung et al. used the SALK_107515 allele (AtMUS81–1) and a second allele (AtMUS81–2) from an independent T-DNA collection (GABI, http://www.gabi-kat.de/) and showed that both produced identical mitotic phenotypes [27]. We used PCR to genotype individuals as homozygous wild type, heterozygous, or homozygous for the insert. Sequence analysis of PCR products from the homozygous insertion line confirmed the T-DNA insertion junction reported in the SALK database. RT-PCR using primers spanning the insertion in lines homozygous for this insertion showed no product (unpublished data). RT-PCR using primers downstream of the insertion showed that transcript levels were greatly reduced in lines homozygous for the insertion (Figure 1B).

### *Atmus81* Mutants Are Sensitive to a Range of DNA-Damaging Agents

**Figure 2:**
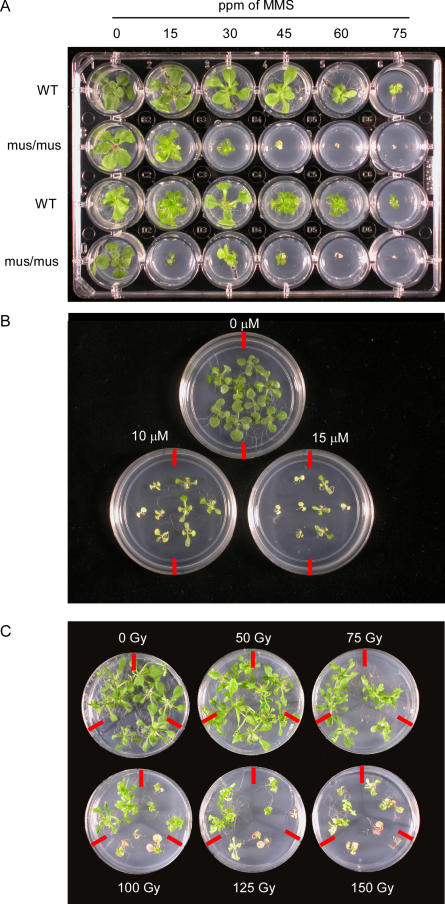
Hypersensitivity of *Atmus81* Mutants to MMS, Cisplatin, and Gamma Radiation (A) Wild-type Col-0 (rows one and three) and mutant *Atmus81*/*Atmus81* (rows two and four) were subjected to a gradient (0–75 ppm) of MMS. The photograph was taken after 20 d. The wild-type plants can grow at each concentration tested while the mutants cannot grow at >30 ppm MMS. (B) Wild-type Col-0 (right side of plate) and mutant *Atmus81*/*Atmus81* (left side of plate) were subjected to various concentrations of cisplatin (0–15 ppm). The photograph was taken after 12 d. Wild-type plants consistently outperformed the mutants at all concentrations tested. (C) Wild-type Col-0 (upper left third), mutant *Atmus81*/*Atmus81* (upper right third), and gamma-hypersensitive mutant *atm-2*/*atm-2* (bottom third) were exposed to various levels of gamma radiation (0–150 Gy). At 75–100 Gy, the *Atmus81* mutants resembled the *atm-2* mutants rather than the wild-type plants.

A feature of *mus81* mutants in other organisms is that they exhibit increased sensitivity to many DNA-damaging agents [19,21,29]. To determine if the *Atmus81* insertion mutant has elevated sensitivity to DNA damage, we exposed seedlings to the radiomimetic MMS. We assayed the growth of both mutant and wild-type individuals (six replicates) on a gradient of MMS concentrations from 0 to 75 ppm (Figure 2A). Visual analysis showed that although both wild-type and homozygous mutants became more sickly with increased concentration of MMS, the *Atmus81* mutants consistently died at 40 ppm while the wild type lines were much healthier at this concentration and survived at even the highest dosages we tested. These results are consistent with those reported by Hartung et al. [27].

To confirm the DNA damage sensitivity phenotype and establish that it was not MMS specific, we conducted a similar assay using cisplatin, which is thought to form interstrand crosslinks and bulky adducts by binding to nitrogen atoms in DNA bases [30]. These adducts subsequently interfere with DNA replication, transcription, and repair [31]. Growth of wild type and *Atmus81* mutants on different concentrations of cisplatin (12 replicates) showed that the mutants have an increased sensitivity (Figure 2B). We also used gamma radiation as a nonchemical source of DNA damage. Consistent with the previous experiments, *Atmus81* mutants (12 replicates) showed increased sensitivity to gamma radiation. Exposure of two-week old seedlings to approximately 100–125 Gy disrupted the growth of the *Atmus81* mutant to an extent similar to the known radiation sensitive mutant *atm-2* [32] which we used as a positive control (Figure 2C). These results demonstrate that the *Atmus81* mutant has increased sensitivity to a range of DNA-damaging agents each with a different mode of action suggesting that *AtMUS81* is active during the somatic cycle and that it has a function in DNA repair.

### AtMUS81 Transcript Levels Increase in Response to DNA Damage

**Figure 3:**
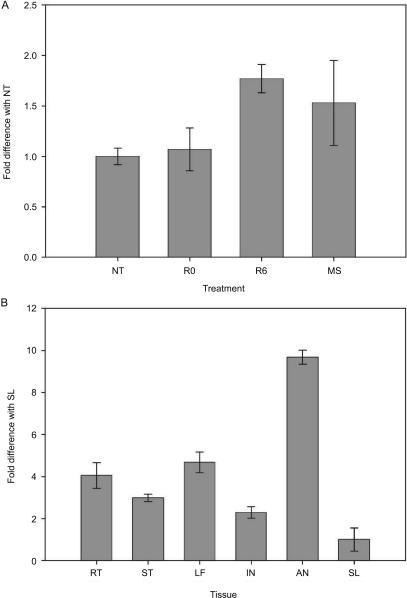
Real-Time qPCR Analysis of *AtMUS81* Transcription (A) RNA from untreated (NT) plants, plants harvested immediately after gamma radiation treatment (R0), plants harvested 6 h after gamma radiation treatment (R6), and plants treated with 50 ppm MMS (MS) was used to measure the induction of the *AtMUS81* transcript. (B) Real-time qPCR analysis of RNA from wild-type root (RT), stem (ST), leaf (LF), inflorescence (IN), anthers (AN), and silique (SL) tissue as a measure of tissue-specific *AtMUS81* expression. The *EF1* gene was used as a control. Error bars are +/− the standard error of the mean calculated from four replicates.

To examine whether the *AtMUS81* transcript was upregulated in response to MMS or gamma radiation treatment we used real-time qPCR. RNA for each experimental treatment was extracted from four 2-wk-old seedlings. Levels of *AtMUS81* transcript were not significantly different among untreated wild-type plants and plants harvested immediately after gamma radiation treatment (125 Gy), but were significantly different after letting the plants recover for 6 h (Figure 3A), indicating transcriptional upregulation in response to DNA damage induced by gamma radiation. The graph shows the mean *AtMUS81* transcription level +/− standard error of the mean from four replicates for each experimental treatment. To assay the levels of *AtMUS81* transcript in response to MMS, 2-wk-old seedlings growing on normal growth media (Murashige and Skoog plates) were transferred to liquid Murashige and Skoog media containing 50 ppm MMS and allowed to incubate for 12 h post transfer. As above, each sample contained RNA extracted from four individuals at the given treatment. Compared to seedlings transferred to liquid media containing no MMS for 12 h, MMS-treated individuals showed a significant increase in *AtMUS81* transcript levels. Fold increase over no treatment control was calculated using the 2^−ΔΔCt^ method [33]. The ubiquitously expressed elongation factor *EF1* was used as a reference gene for this analysis [34,35].

### *AtMUS81* Transcript Levels Are Upregulated during Meiosis

We have hypothesized that *AtMUS81* is active during meiosis. To test this hypothesis, we used real-time quantitative PCR (qPCR) to assess the levels of *AtMUS81* transcript in various tissue types (Figure 3B). *AtMUS81* transcript levels were measured relative to transcript levels of the elongation factor *EF1,* which was used as a reference gene. *EF1* is expressed equally in all tissues and has been used for this purpose in other quantitative analyses of tissue specific transcript levels [34,36]. Roots, stems, leaves, and inflorescences showed consistent moderate levels of expression. In contrast, the expression levels in anthers were considerably higher. Using the levels in the silique, which showed the lowest levels of *AtMUS81* transcript as a reference point set at 1.0, the transcript levels in the anthers were increased 9.68 +/− 0.33-fold.

### Pollen Viability Is Decreased in the *Atmus81* Mutant

**Figure 4:**
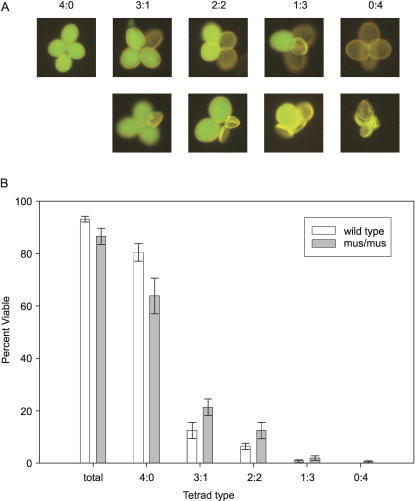
Pollen Viability in Wild Type and *Atmus81* Mutants (A) Pollen tetrads from plants homozygous for three different fluorescent markers were examined. Pollen was classified as nonviable if grains were aborted (bottom row) or if all of the fluorescent proteins were not expressed (top row). (B) The *Atmus81* mutant has lower levels of pollen viability. Viability of wild type (open bars) is compared to *Atmus81*/*Atmus81* plants (gray bars) and is also broken into tetrad categories (4:0, 3:1, 2:2, 1:3, 0:4; viable:nonviable). Error bars are +/− the standard error of the mean.

Other A. thaliana meiotic genes such as *RAD51*, *MND1*, *MEI1,* and *SPO11* exhibit decreased pollen viability when disrupted, either as a result of chromosome fragmentation or segregation defects [37–39]. This is also true in many other organisms, as a failure to recombine at wild-type levels often leads to gametic abnormalities. To measure pollen viability in the *Atmus81* mutant, we monitored production of dsRED, YFP, and CFP fluorescent proteins in the pollen tetrads that were homozygous for all three markers (Figure 4A). Pollen grains were scored as nonviable when they were morphologically aberrant (small and misshaped) or when all three color markers failed to express (Figure 4A). In our hands, this fluorescent marker–based assay for pollen viability is more robust than staining procedures (e.g., flourescein diacetate, propidium idodide, or Alexander's stain), producing more consistent results [40]. In contrast to other published work [27], we found that the *Atmus81* mutant shows a statistically significant decrease in overall pollen viability, 87% (788 tetrads) versus 96% in wild type (853 tetrads; Figure 4B). We also found that the *Atmsh4* mutant, which has been previously reported to have a pollen viability defect [12] does indeed exhibit high levels of pollen lethality with a preponderance of 2:2 viable:nonviable tetrads (unpublished data), common for chromosome segregation defects and a hallmark of many meiotic mutants in S. cerevisiae.

### *AtMUS81* Is Essential for a Subset of Meiotic COs

**Figure 5:**
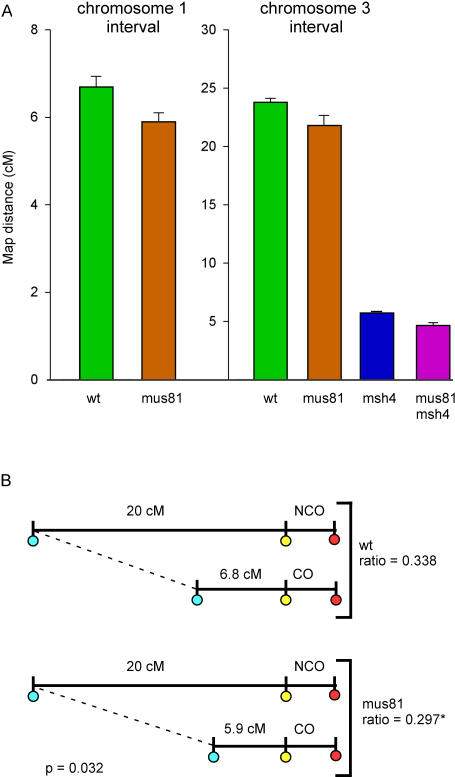
Meiotic Recombination in *Atmus81* and *Atmsh4* Mutants (A) Characterization of the meiotic recombination phenotype using intervals on Chromosomes 1 (left) and 3 (right). Green bars represent pooled data from wild type and heterozygotes, orange bars represent *Atmus81* mutants, blue bars represent *Atmsh4* mutants, and purple bars represent *Atmus81*/*Atmsh4* double mutants. Error bars are +/− the standard error of the mean. (B) Interference analysis of the *Atmus81* mutant using three linked markers on Chromosome 5. Each pair of graphs (wild type and heterozygotes top; *Atmus81* bottom) shows the genetic distances of an interval without and with an adjacent CO. The ratios of these genetic distances with adjacent CO: without a CO were significantly different between the pooled wt/heterozygotes and *Atmus81* mutants with a one-tailed *p-*value of 0.032 (see Materials and Methods for calculation of the *p*-value).

The elevated *AtMUS81* transcript levels in meiotic tissues and the decreased pollen viability in the *Atmus81* mutant are both consistent with a role in meiotic recombination. To assess this role, we characterized the meiotic defects in both *Atmus81* and *Atmsh4* by measuring meiotic CO levels. To facilitate the use of tetrad analysis, we crossed mutant *Atmus81* and *Atmsh4* individuals into the *qrt* tetrad-producing background. *Atmus81*/*Atmus81; qrt1–2*/*qrt1–2* or *Atmsh4*/+*; qrt1–2*/*qrt1–2* plants were then crossed to lines that were homozygous for fluorescent marker genes flanking a genetic interval. Initially, intervals on Chromosome 1 (6.7 cM) and Chromosome 3 (23.7 cM) were used (Figure 5A). These intervals were previously described in Francis et al. [15]. F1 progeny from these crosses that were heterozygous for *Atmus81* or *Atmsh4* and heterozygous for the fluorescent interval were allowed to self and marker/+ individuals were scored in the F2 generation for recombination. Recombination in these individuals was scored in the pollen tetrads before the genotypes of the individuals were determined (blind scoring). After all recombination data had been collected, the plants were PCR genotyped for the *Atmus81* and *Atmsh4* T-DNA insertions. The *Atmus81* and *Atmsh4* mutant alleles segregated in this generation such that the homozygous mutant:heterozygote:wild-type ratio was approximately 1:2:1, as expected.

The resulting collection of tetrad recombination data was analyzed using the Perkins mapping function of *X* = 100[(1/2*T* + 3NPD)/*n*] [41]. The *Atmus81* mutants showed a ∼12% reduction in genetic distance in the interval on Chromosome 1 (12,323 tetrads) and a ∼9% reduction in the interval on Chromosome 3 (6,710 tetrads; Figure 5A). The *Atmsh4* mutant showed a ∼75% reduction in genetic distance in the Chromosome 3 interval (188 tetrads). *Atmus81;Atmsh4* double mutants showed a 80% decrease in genetic distance (408 tetrads) compared to wild type, which was the greatest reduction observed and was significantly different from either of the single mutant distances. The genetic distance in the double mutant was further lowered (4.7 cM to 4.5cM) if we included data from three-member tetrads, in which one can infer the marker genotype of the fourth member of the tetrad. Interval 1 analysis was not conducted for the *Atmsh4* mutant.

### The Remaining COs of the *Atmus81* Mutant Are Interference Sensitive

The observation that CO levels are reduced in *Atmus81* plants is consistent with *AtMUS81* playing a role in an interference-insensitive CO pathway [6]. This view would predict that interference would still be intact in the remaining COs in these mutants. To test this prediction we crossed *Atmus81*/*Atmus81*; *qrt1–2*/*qrt1–2* lines to a line with three linked inserts on Chromosome 5 (also in the *qrt1–2* background), each encoding a different color fluorescent protein (Figure 5B). These inserts define two adjacent intervals, which could be simultaneously assayed for recombination. This enabled us to do a type of interference analysis in which we measured genetic distances with and without the presence of a simultaneous event in the neighboring interval, a type of analysis that has been applied to S. cerevisiae tetrad data [7,42]. The ratio of genetic distance with the presence of an adjacent CO relative to the distance when an adjacent CO is absent gives a value that is similar to but distinct from the coefficient of interference.

Within the smaller of the two adjacent intervals, we observed a genetic distance of 20.0 cM and 6.8 cM without and with an adjacent CO, respectively, in data pooled from wild type and *Atmus81/+* heterozygotes (12,718 tetrads). In the *Atmus81* mutant, we observed distances of 20.0 cM and 5.9 cM, without and with an adjacent crossover, respectively (5,905 tetrads). A statistical comparison of wild type and *Atmus81* mutants was significantly different with a one-tailed *p-*value of 0.032 when we compared ratios of genetic distances in the interval with and without an adjacent CO (Figure 6B). This is consistent with the hypothesis that *AtMUS81* is involved in a subset of COs that are interference insensitive.

## Discussion

In order to understand the role of *AtMUS81* in DNA-damage repair, we examined the specificity of the defect using agents with different mechanisms of action for producing genotoxic damage. In a previous study by Hartung et al., *Atmus81* mutants were shown to be sensitive to mitomycin C and MMS but were not sensitive to the double-strand break–inducing agent bleomycin [27]. Our results demonstrate sensitivity of *Atmus81* mutants to cisplatin, which creates bulky adducts as well as MMS and gamma radiation, each of which produce a wide range of DNA damage including double-strand breaks (gamma radiation). This phenotype can be explained by a role for *AtMus81* in repairing collapsed replication forks, a somatic repair role postulated for *MUS81* in S. cerevisiae [25], but it does not rule out a role in other kinds of damage repair.

Our result does demonstrate the nonredundancy of the *AtMUS81* gene (At4g30870) in A. thaliana. Build 6.0 of the A. thaliana genome in the NCBI database (http://www.ncbi.nlm.nih.gov/gquery/gquery.fcgi) includes another annotated *MUS81-like* (At5g39770). This sequence is most likely a nonfunctional pseudogene, as the putative protein has three in-frame stop codons in two different exons [27]. A likely promoter insertion line with a T-DNA insertion 554 bp (SALK_051926) upstream of the first predicted exon did not show sensitivity to MMS at any concentration compared to wild type (unpublished data). Hartung et al. report no success in trying to amplify even partial cDNA from this predicted gene [27].

Looking at two different genetic intervals on Chromosomes 1 and 3, we observed an average 10% decrease in meiotic crossing over in the *Atmus81* mutant background. This result implies that AtMus81 has a meiotic role and is involved in the processing of a subset of meiotic COs. This meiotic role is consistent with the observed decrease in pollen viability. Our qPCR result showing that *AtMUS81* transcript levels are increased by over 9-fold in the anthers is also consistent with this view. The *Atmus81;Atmsh4* double mutant showed a larger meiotic recombination defect than either of the two single mutants, which demonstrates independence of the two pathways. However, the fact that residual crossovers still remain in this mutant combination suggests that there is a third pathway in A. thaliana that can produce COs in the absence of At*Mus81* and At*Msh4*. A similar small residual amount of COs were also observed in the *S. cerevisiae mus81*;*msh4* double mutant [22].

Previous statistical modeling of interference in A. thaliana suggested that, on a genome-wide level, a small fraction of COs in A. thaliana should be free of interference and thus randomly distributed [6,14]. The modest reduction that we observed in the *Atmus81* mutant in two intervals is consistent with our hypothesis that *AtMUS81* is a mediator of the secondary interference-free pathway. Interference measurements in *Atmus81* plants confirmed that not only were the remaining crossovers interference sensitive, but the overall levels of interference were significantly increased in the mutant background. This result suggests that like *S. cerevisiae, A. thaliana* has interfering COs coexisting with noninterfering COs. In *S. cerevisiae,* noninterfering *MUS81*-mediated COs are thought to be ∼30% of the total [8], while our result shows ∼10% of *AtMUS81* mediated noninterfering COs in the two intervals we examined in A. thaliana.

Other methods of assaying recombination in A. thaliana have been developed, and one in particular involving a tandem disrupted beta-D-glucuronidase (GUS) gene [43] was recently applied to study recombination in *Atmus81* mutants [27]. Hartung et al. used a GUS reporter construct to assay inter- and intrachromosomal recombination and found no significant difference between wild type and *Atmus81* mutants. Interestingly, it was observed that mitotic recombination was decreased in the *Atmus81* mutant after bleomycin treatment. These results are compatible with the meiotic defect observed in this study. Events producing a functional GUS construct arise either by unequal exchange or intrachromosomal recombination and thus do not measure simple meiotic COs. Hartung et al. state that the COs postulated to exist in the secondary recombination pathway [6,12] would be undetectable using their GUS reporter assay [27]. Our fluorescent system allows us to visualize true homologous meiotic COs and provides the opportunity to generate large datasets with relative ease [15]. These large datasets also make possible the ability to detect small differences between mutant and wild-type lines.

A recent result from S. pombe suggests that in this organism, in which all COs are mediated by Mus81-Eme1, the predominant meiotic recombination intermediate is a single Holliday junction (sHJ) [26]. This is in contrast to data from S. cerevisiae showing that the predominant meiotic recombination intermediate is a dHJ [44]. Intriguingly, these dHJs have been reported to coexist with a significant number of sHJs [45]. This leads to the possibility that the two pathways are biochemically distinct in that *MSH4/MSH5* mediated COs go through a dHJ intermediate while *MUS81* mediated COs go through a sHJ intermediate, an idea recently proposed by Cromie et al [26]. Our result shows that in *A. thaliana, AtMSH4* and *AtMUS81* mediate distinctly different CO generating pathways. It is certainly possible that in this organism, these two pathways go through biochemically distinct intermediates, which could be double and single HJs, respectively. Direct detection of physical recombination intermediates using 2-D electrophoresis or electron microscopy in A. thaliana may help determine the structure of recombination intermediates in this two pathway organism.

Mus81p typically forms a functional heterodimer with a second protein in vivo. [20]. We have run an iterative BLAST search (PSI-BLAST; http://www.ncbi.nlm.nih.gov/BLAST/) on the A. thaliana protein database using the published protein sequences of the known *MUS81* interactors from S. pombe (Eme1p) and *S. cerevisiae* (Mms4p) in an attempt to find the A. thaliana homolog of the *MUS81* interactor: *AtEME1*. Our search has resulted in two candidates for *AtEME1:* At2g21800 (4e−21) and At2g22140 (1e−13). These genes are closely linked together at a physical distance of ∼100 kb. We are working to identify and characterize all mutant combinations of *Ateme1* candidates as well as confirm a direct interaction with AtMus81.

## Materials and Methods

### Plant and growth conditions.

The *qrt1–2* (Columbia-3 background) line was kindly provided by Daphne Preuss (The University of Chicago). The SALK_107515 *(Atmus81)* and SALK_136296 *(Atmsh4)* lines were obtained from the Arabidopsis Biological Resource Center at The Ohio State University (http://www.arabidopsis.org/). Seeds were sown on either Pro-mix (Premier Horticulture, http://www.premierhort.com/) or Metromix-400 (Sun-Gro, http://www.sungro.com/) and stratified for 3–4 d at 4 °C. In each experiment, the same soil type was used. Plants were germinated and grown under long day conditions (18h day 6h night) at 20 °C. Temperatures were monitored with thermometers on the same shelves where the plants were grown. Seeds from plants used in the DNA sensitivity assays were surface sterilized in 10% bleach with 0.1% Triton X-100and grown on either 24-well tissue culture plates (Fisher Scientific, https://new.fishersci.com/) or on 60-mm Petri dishes in MS media with 5.0 g Phyto agar/L (Research Products International, http://www.rpicorp.com/). All parental strains are available from the Arabidopsis Biological Resource Center at Ohio State University (Columbus, Ohio, Unites States). All fluorescent tag lines are available on request from G. P. Copenhaver.

### DNA extraction and PCR analyses.

Genomic DNA was extracted from 2–3 cauline leaves as described by Copenhaver et al. [46]. PCR (30 cycles) was used to identify plants that were homozygous and heterozygous for the two T-DNA insertions, SALK_107515 *(Atmus81)* and SALK_136296 *(Atmsh4)*. For the SALK_107515 line the wild-type allele was amplified using SALK_107515F (see Table S1 for oligonucleotides used in this study) and SALK_107515R while the mutant allele was amplified using LBB1, a primer specific to the left border, and SALK_107515R. For the SALK_136296 line, the wild-type allele was amplified using SALK_136296F and SALK_136296R while the mutant allele was amplified using LBB1 and SALK_136296R. The mutant allele products were purified and sequenced to confirm their identity. The *atm-2* mutant was the same as characterized by Garcia et al. and homozyogous mutant plants were identified using the primers LBa1 and ATM126 [32].

### Mutagen assays.

*MMS:* wild type and homozygous *Atmus81* mutants were surface sterilized and plated directly in 24-well tissue culture plates (1 ml/well) containing solid MS media with the respective concentration of MMS (0–75 ppm). Photos were taken at 14 d after plating.

*Cisplatin:* wild type and homozygous *Atmus81* mutant were surface sterilized and plated directly on 60-mm Petri dishes containing solid MS media (15 ml/plate) with the respective concentration of cisplatin (0–25 ppm). Photos were taken at 12 d after plating.

*Gamma radiation:* wild type, homozygous *atm-2* (positive control) [32], and homozygous *Atmus81* mutant were surface sterilized and plated directly on 60-mm Petri dishes containing solid MS media. At 7 d, these plates were placed in a Gammator Cesium-135 irradiator (Radiation Machinery Corporation, Parsippany, NJ) for times corresponding to the appropriate dosages. Photos were taken 13 d after removal (20 d total).

### RNA isolation, AtMUS81 cDNA analysis, and real-time qPCR.

All RNA used in this study was isolated using the RNeasy Plant Mini Kit (Qiagen, http://www.qiagen.com) according to the manufacturer's protocol. RT-PCR was performed using the Thermoscript cDNA synthesis kit (Invitrogen, http://www.invitrogen.com) according to manufacturer's protocol using ∼1 ug of RNA and maximum incubation times. RNA used for confirmation of published cDNA sequence was isolated from young inflorescence tissue. RACE (5′ and 3′) was performed using the Takara 3′ and 5′ RACE core kits using manufacturer's protocol (Takara, http://www.takara-bio.com/) using ∼1 ug of RNA and maximum incubation times. Primer sets used to confirm the published sequence of the cDNA were M81_ALB1F, M81_ALB2R, PS3 and PR3 [27]. The 3′ RACE forward primer was M81S1_F and the 5′ RACE primers used were M81RT/Phos, S1, S2, A1, and A2. Analysis of transcription levels in the *Atmus81* mutant was conducted using RT-PCR as above with the following primer sets: M81S1_F and M81S1_R, M81S2_F and M81S2_R, and oMC571 and oMC572 [47] (APT1 control).

*Real-time qPCR:* To test *AtMUS81* transcript levels after gamma radiation, two-wk-old seedlings growing on 60-mm solid MS media plates were placed in a Gammator as above and were allowed to receive a dosage of 125 Gy. RNA was isolated from these plants directly after removal from the radiation source as well as 6 h after removal. Treated samples were compared to plants grown in the exact same manner that were not subject to radiation. To test *AtMUS81* transcript levels after MMS treatment, two-wkold seedlings were transferred either to liquid MS media containing 50 ppm MMS (treated) or 0 ppm MMS (untreated control) and allowed to incubate for 12 h post transfer. qPCR was conducted on these samples as above. Tissue-specific transcription analysis was conducted using ∼100 mg each of root, stem, leaf, young inflorescence, anther, and silique for RNA preparation.

First-strand cDNA synthesis was conducted using 2 μl of RNA sample with Superscript III system according to the manufacturer's protocol (Invitrogen). A 40-cycle real-time PCR reaction with optical reads after each cycle was performed on the Opticon real-time thermal cycler (MJ Research, http://www.bio-rad.com/) with the SYBR Perfect real-time qPCR premix (Takara) according the manufacturer's protocol. The specificity of the reaction was determined using a melting curve from 60 °C to 95 °C with reads every 0.2 °C. Opticon 3 software (MJ Research) was used as the interface for execution and initial analysis. Reaction sizes were 25 μl containing 2 μl of undiluted cDNA. Specific primer sets for detection of *AtMUS81* transcript were M81RTRT3_F and M81RTRT3_R, which produces a 189-bp fragment. The results obtained were standardized to the constitutive EF1A4α gene expression level [34] and amplified with EF1F and EF1R, which produces a 73-bp fragment. Efficiencies of the different primer sets were determined by dilution sets to be equal. The 2^−ΔΔCt^ method [33] was used to quantify fold increase of *AtMUS81* transcript. DNA damage assays were compared relative to no-treatment controls and the tissue specific samples were compared to silique, which had the lowest levels of *AtMUS81* transcript and was set at 1.0 for the purpose of relative comparison. For all assays, samples were run in quadruplicate and standard error of the mean increase was calculated.

### Microscopy.

Segregation patterns of fluorescent alleles in pollen tetrads were measured by using a Nikon (http://www.nikonusa.com/) E1000 epifluorecence microscope equipped with filters from Chroma Technology (http://www.chroma.com/). Pollen was collected by dipping flowers into a 10 μl drop of PGM media (34% sucrose, 4 mM CaCl_2_, 3.25 mM boric acid, 0.1% Triton-X, pH 7.5) on a glass slide with coverslip. Pollen viability was assayed by monitoring the production of dsRED, YFP, and CFP fluorescent proteins in pollen tetrads in the homozygous interval 5 background (FTL_1273; FTL_1659; FTL_993). All photographs were taken using a Nikon Coolpix5000 color digital camera. Figures were prepared using either Photoshop (Adobe, http://www.adobe.com) or Canvas (Deneba, http://www.deneba.com/).

### Linkage and statistical analysis.

See Table S2 for a list of the color and chromosomal position of all fluorescent transgenic markers used in this study. To measure the genetic distance between any two transgenic markers, tetrads were designated parental ditype, nonparental ditype, or tetratype, depending on the segregation of the marker pair. Map distances were then calculated by using the Perkins formula: *X* = 100[(1/2*T* + 3NPD)/*n*] [41]. Interference was measured by measuring CO frequencies in adjacent intervals and dividing the tetrad data for one interval into groups based on the presence or absence of a CO in an adjacent reference interval [7]. If the genetic distance in the interval in question is significantly lower with the presence of a CO in the adjacent interval, we conclude interference extending from one interval to the other [7,42]. For a pair of adjacent intervals, we compared interference between datasets (wild type and mutant in this case). We calculated the ratio of genetic distances by dividing distance in an interval with an adjacent CO by the distance without the presence of an adjacent CO. These ratios were statistically compared by obtaining a *Z*-score using the following equation:

*Z* = |*R*1−*R*2|/√[var (*R*1−*R*2)], where *R*1 is the ratio in wild type and *R*2 is the ratio in the mutant.

The significance of the difference between these two ratios was assessed using a one-tailed test as described on the Stahl Lab Online Tools (http://molbio.uoregon.edu/~fstahl).

## Supporting Information

Table S1 Sequence Information for Oligonucleotides Used (45 KB DOC)

Table S2 Fluorescent Marker Inserts Used in This Study (32 KB DOC)

## Abbreviations

CO: crossover
dHJ: double Holliday junction
GUS: beta-D-glucuronidase
HJ: Holliday junction
sHJ: single Holliday junction
MMS: methyl methanesulfonate
qPCR: quantitative PCR
RACE: rapid amplification of complementary ends
RT-PCR: reverse-transcriptase PCR
T-DNA: transfer DNA
