# Supplementary material for: The Role of AtMUS81 in Interference-Insensitive Crossovers in A. thaliana
Source: PLoS Genet. 2007 Aug 10;3(8):e132. doi: 10.1371/journal.pgen.0030132 (PMC1941751; doi:10.1371/journal.pgen.0030132)
Supplement: Supplementary file 1 [file pgen.0030132.st001.doc]

Supplemental Table 1. Sequence information for oligonucleotides used

| Primer Name | Sequence (5’-3’) |
| --- | --- |
| SALK_107515_LP (M81_F) | gacagttgaaggtcgggaag |
| SALK_107515_RP (M81_R) | aattttccacaaaccctttgg |
| LBb1 | gcgtggaccgcttgctgcaact |
| SALK_136296_LP | tggaatggatcaatgagttcc |
| SALK_136296_RP | aatcggtgagtcaggtttcag |
| oMC571 (APT1_F) | tcccagaatcgctaagattgcc |
| oMC572 (APT1_R) | cctttcccttaagctctg |
| LBa1 | gcgtggaccgcttgctgcaact |
| ATM126 | tctctccttgtttcaagctctgc |
| M81S1_F | acaaatattccacgcctccctcct |
| M81S1_R | tcctcagcacatcaaatccctcca |
| M81S2_F | tgctcggcataagtaccttgagac |
| M81S2_R | tgccggtttgctctggtcattatc |
| PS3 | gacttggacactctaagag |
| PR3 | aagcaagagacaaaagcgttg |
| M81_ALB1F | tcccaatcagcaaggaattt |
| M81_ALB2R | cgaacaggtgggattttgat |
| M81RT-Phos | -/5Phos/atcaataagctcttg |
| S1 | ccgcaaagaaattcagtggg |
| S2 | acagaaggactacaaatggg |
| A1 | ttcccagcacgattatcagg |
| A2 | cttgctcagaactttcaaca |
| M81RTRT3_F | tctctgtaatgtgccatctt |
| M81RTRT3_R | ttgaacctgatgaacatgga |
| EF1_F | ctggaggttttgaggctggtat |
| EF1_R | ccaagggtgaaagcaagaaga |
