# Supplementary material for: The Role of AtMUS81 in Interference-Insensitive Crossovers in A. thaliana
Source: PLoS Genet. 2007 Aug 10;3(8):e132. doi: 10.1371/journal.pgen.0030132 (PMC1941751; doi:10.1371/journal.pgen.0030132)
Supplement: Supplementary file 2 [file pgen.0030132.st002.doc]

Supplemental Table 2. Fluorescent marker inserts used in this study

| Name | Type | Chromosome_position |
| --- | --- | --- |
| FTL_992 | CFP | I_9,850,022 |
| FTL_1313 | dsRED | I_11,130,549 |
| FTL_1500 | CFP | III_498,916 |
| FTL_1371 | dsRED | III­_4,319513 |
| FTL_1273 | dsRED | V_18164269 |
| FTL_1659 | YFP | V_23080567 |
| FTL_993 | CFP | V_25731311 |
